# Efficacy and safety of acupuncture in patients with cancer-related fatigue: A protocol for systematic review and meta-analysis

**DOI:** 10.1097/MD.0000000000022759

**Published:** 2020-10-16

**Authors:** Tai-Jun Jiang, Feng-Ya Zhu, Li-Jie Tang, Zheng-Kang Liu, Xi Wu

**Affiliations:** Acupuncture and Tuina School, Chengdu University of Traditional Chinese Medicine, Chengdu, Sichuan, China.

**Keywords:** acupuncture, cancer-related fatigue, protocol, systematic review

## Abstract

**Background:**

Cancer-related fatigue (CRF) is considered a common complication of cancer or cancer treatment, which has a serious adverse effect on the life and of cancer patients, leading to a decline in their quality of life (QoL). The existing clinical trials revealed that acupuncture has a positive effect on CRF, and there are fewer adverse events confirmed in the corresponding systematic review. However, in recent years, new studies on using acupuncture to treat CRF were conducted, so in order to evaluate its efficacy, an updated systematic review. This protocol provides research methods for systematic review and meta-analysis of the safety and effectiveness of acupuncture for the treatment of CRF.

**Methods:**

We will searched the randomized controlled trial literature of acupuncture treatment for CRF in 4 English and 4 Chinese databases, including PubMed, MEDLINE, Web of Science, Cochrane Central Register of Controlled Trials (Central), China National Knowledge Infrastructure (CNKI), China Biomedical Literature Database(CBM), China Science Journal Database (VIP), and Wanfang Database. Simultaneously, other resources are manually retrieved which include reference lists of identified publications, conference articles, and grey literature. We also included the clinical randomized controlled trials of acupuncture treatment for CRF in the study. The search language is limited to Chinese and English. Two trained reviewers independently completed research screening, data extraction, and research quality assessment. RevMan (V.5.3) software was used to perform data statistical analysis and Grading of Recommendations Assessment, Development, and Evaluation (GRADE) was used to evaluate the quality of evidence.

**Results:**

This study is based on past and present clinical evidence to comprehensively evaluate the effectiveness and safety of acupuncture treatment for CRF.

**Conclusion:**

Through this systematic review, we will provide the latest high-quality evidence of whether acupuncture treatment for CRF is effective and safe and also provide a theoretical basis for clinicians to choose acupuncture for the treatment of CRF.

**Systematic Review Registration:**

INPLASY 202090049.

## 1 Introduction

Fatigue is considered a common symptom in cancer patients. The U.S. National Comprehensive Cancer Network defines cancer-related fatigue (CRF) as a distressing, persistent, subjective sense of physical, emotional, and/or cognitive tiredness or exhaustion related to cancer or cancer treatment that is not proportional to recent activity and interferes with usual functioning. Currently, the prevalence of CRF is about 50% to 90%.^[1]^ Moreover, different cancer types, evaluation criteria,^[2,3]^ treatment stages, and genders have different prevalence rates.^[4]^ Some studies have shown that the incidence of CRF is closely related to skeletal muscle and mitochondrial dysfunction, peripheral immune activation and inflammatory dysfunction, CNS disorders,^[5]^ as well as concomitant diseases (anemia, cardiac conditions, diabetes mellitus, etc.) and cancer treatment, psychological factors of patients,^[6]^ psychological factors of patients, and so on. However, CRF can arise at any stage of cancer and tends to increase during and after treatment, which has a serious adverse effect on patients quality of life (QoL).^[7,8]^ CRF has become a public health problem considering the increased medical and social costs. Therefore, how to treat CRF economically and effectively and improve the QoL of CRF patients has become an urgent problem to be solved.

Nowadays, we do not have a unified method for the treatment of CRF, but we have many CRF intervention measures which include physical exercise, pharmacological treatments, nutraceutical treatments, psychological intervention, cognitive behavioral therapy, art therapy, and complementary alternative therapy.^[9–11]^ However, there are corresponding limitations of these interventions including the commonly used drug methylphenidate (MPH), which is a traditional psychoactive drug. From the related research reports, it is clear that MPH can significantly improve CRF, but it causes side effects such as headaches, dizziness, anxiety, nausea, dry mouth, and other adverse events.^[12,13]^ Also, using erythropoiesis-stimulating agents (ESAs) is another commonly used drug treatment as they stimulate the rise of hemoglobin to improve the anemia caused by chemotherapy in CRF patients and improve the symptoms related to fatigue.^[14]^ However, recently, researchers have found that using ESAs to treat CRF has increased the risk of developing venous thrombosis.^[15–17]^ Simultaneously, there are also other studies which have shown that drug intervention has not improved CRF.^[11]^ However, there is no unified conclusion on whether other interventions such as exercise, psychological intervention,^[18,19]^ and cognitive behavioral therapy can improve CRF as the effect is not obvious.^[20]^

Acupuncture may cause soreness, numbness, distension, and heaviness due to piercing acupuncture needles into specific places (acupuncture points) of the body and this is called Deqi which is necessary to achieve a specific therapeutic effect. Moreover, acupuncture is considered one of the most important components of traditional Chinese medicine and it is characterized by simplicity, effectiveness, and safety. The use of acupuncture has expanded in recent years to include the treatment of various diseases in the world, such as migraine,^[21]^chronic stable angina,^[22]^ obesity,^[23,24]^ and postprandial distress syndrome.^[25]^ Therefore, the researchers focused on whether acupuncture can improve cancer-caused diseases. Studies have proved that acupuncture is a safe and effective treatment for CRF, which can improve the CRF and improve their QoL.^[26–31]^ However, there are differences between the results of various studies on the safety and effectiveness of acupuncture for the treatment of CRF in different periods. Therefore, it is necessary to summarize the latest researches on acupuncture for the treatment of CRF and design the corresponding systematic review and meta-analysis to obtain the latest results to assess the safety and effectiveness of acupuncture for the treatment of CRF.

## 2 Methods and analysis

### 2.1 Registration of the review

The protocol of this systematic review has been registered on INPLASY with a registration number 202090049 and the reporting of this protocol has been structured according to the preferred reporting items for systematic reviews and meta-analyses protocols (PRISMA-P) statement guidelines.^[32]^ The PRISMA guidelines and the Cochrane Handbook were followed to evaluate the study of inclusion.

### 2.2 Ethics

This systematic review aims to evaluate the effectiveness and safety of acupuncture for the treatment of CRF through the published clinical randomized controlled trials, so no ethical approval is required.

### 2.3 Inclusion and exclusion criteria

#### 2.3.1 Type of study

This study only included randomized controlled trials, and we excluded any other types of studies, such as other nonrandomized controlled trials, case reports, animal experiments, reviews, and secondary analysis.

#### 2.3.2 Type of participants

This study included subjects who were cancer patients diagnosed through clinical and laboratory examinations (including but not limited to gender, age, race, and education level) and met the diagnostic criteria of CRF. Subjects who were diagnosed with other fatigue diseases like chronic fatigue syndrome, cirrhosis, AIDS, rheumatic and chronic inflammatory diseases, multiple sclerosis, and so on were excluded.

#### 2.3.3 Types of interventions

This study included different types of intervention in the treatment group such as acupuncture (hand acupuncture or electroacupuncture) or acupuncture combined with other therapies; on the other hand, the control group was waiting for treatment or adopting other treatment methods. However, when the intervention in the treatment group is acupuncture combined with other treatments, the corresponding control group should use the same other treatments.

#### 2.3.4 Types of outcome measures

##### 2.3.4.1 Primary outcome

This is the cancer-related fatigue scale score.

##### 2.3.4.2 Secondary outcomes

This will include:

1. TCM Syndrome Integral Scale
2. Adverse event

### 2.4 Data sources

Four English and 4 Chinese databases, including PubMed, MEDLINE, Web of Science, Cochrane Central Register of Controlled Trials (Central), China National Knowledge Infrastructure (CNKI), China Biomedical Literature Database (CBM), China Science Journal Database (VIP), and Wanfang Database were searched electronically, and we searched the databases to June 31, 2020. However, due to the researchers language limitations, the search language is Chinese and English. Besides, other resources are manually retrieved which include reference lists of identified publications, conference articles, and grey literature. The searching strategy of PubMed is presented in Table 1.

**Table 1:**
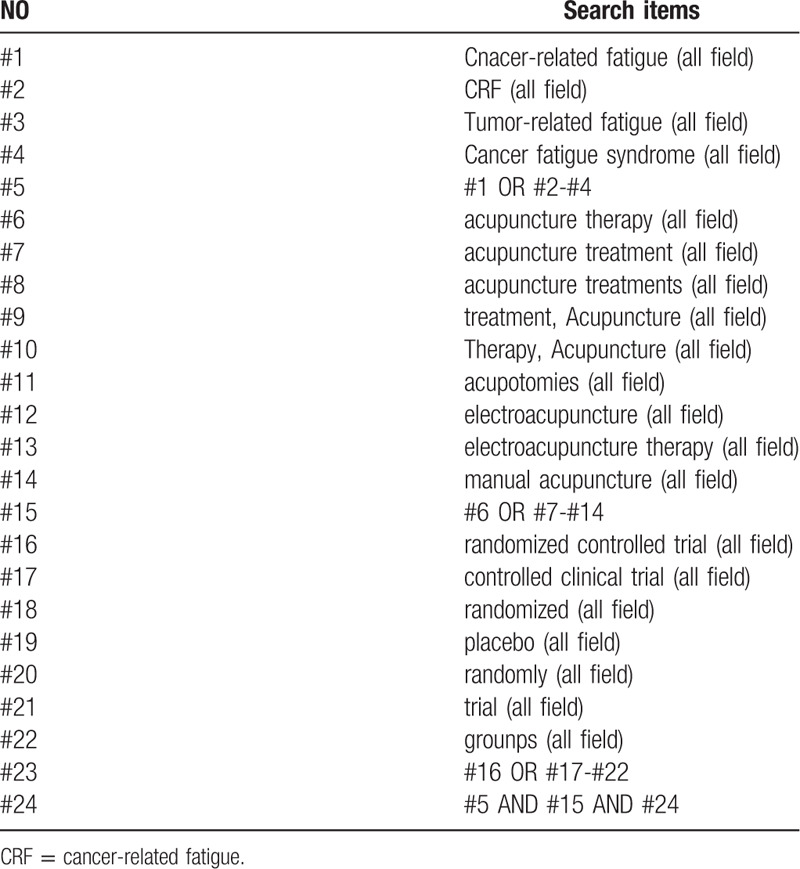
Search strategy used in Pubmed.

### 2.5 Selection of studies

**Figure 1:**
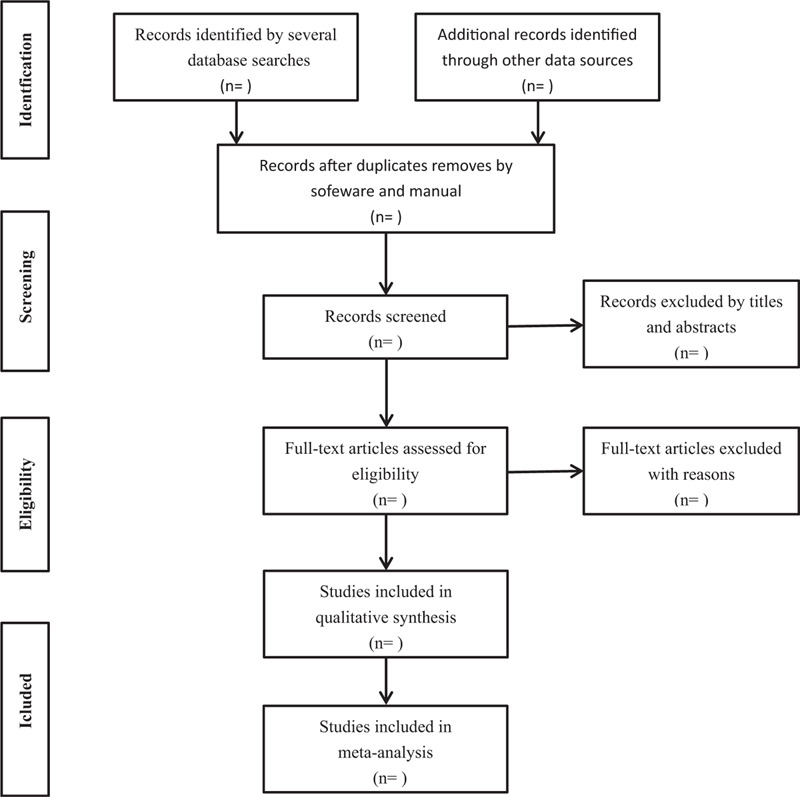
Flow chart of study selection.

After all the documents have been retrieved, the search results were imported into EndNote software (V.X9) to eliminate duplicate imported documents. Two trained reviewers (TLJ and LZK) independently screened qualified documents by title and abstract. In the next analysis, the 2 reviewers cross-checked the documents, and disagreements in the literature screening, if any, were resolved through discussion. After that, if there are still differences, the third reviewer (ZFY) will make the decision. The flow process of filtration is shown in a PRISMA flowchart in Figure 1.

After the screening, if the number of the documents included is more than 3, then a meta-analysis will be conducted, but if it is less than 3, then a descriptive analysis will be conducted.

### 2.6 Data collection and management

The corresponding data extraction form, was designed, and the data were extracted by 2 reviewers (JTJ and TLJ) independently. After the extraction, they were cross-checked. Moreover, any differences were discussed and resolved by all reviewers. The data extraction table includes the following: first author, title, country, year of publication, source database, participants (gender, country, average age, cancer type, participants Western medicine treatment before intervention, etc.), trial characteristics (number of groups, the sample size of the intervention group and control group, random method, blind method, method of acupuncture, frequency of treatment, course of treatment, acupuncture points, duration of acupuncture treatment, etc.), primary outcomes (scores of cancer-related fatigue-related fatigue scale), secondary outcomes (Chinese medicine syndrome scores, adverse events, etc.), follow-up time, and conflicts of interest.

### 2.7 Assessment of risk of bias in the included studies

The “deviation risk” tool in Cochrane Handbook v.5.1.0 is used to assess the deviation risk of each article in the literature. This assessment includes sequence generation, allocation sequence hiding, the blindness of participants, personnel, and outcome evaluators, incomplete outcome data, selective outcome reporting, and other sources of bias. The assessment results are classified into 3 levels, that is, low risk, high risk, and uncertainty risk. If the risk of bias is high in the literature, then we will try to explain and discuss the causes of bias.

### 2.8 Assessment of heterogeneity

*I*^2^ and Chi-Squared tests are used to evaluate the heterogeneity of the included trials. When the heterogeneity was not obvious (*P* > .1 and *I*^2^ < 50%), the fixed-effect model was used, and when the heterogeneity was significant (*P* < .1 and *I*^2^ > 50%), the random-effect model was utilized.

### 2.9 Assessment of reporting biases

The assessment of the reporting bias is performed using the funnel chart and Eggers method;^[33]^ that is, when the number of the studies included exceeds 10 trials, we will use the funnel chart to assess reporting biases and use Eggers method to test funnel chart asymmetry. A symmetrical funnel plot indicates a low risk of bias. On the other hand, the greater the asymmetry in the funnel chart, the higher the risk of bias.

### 2.10 Data synthesis

RevMan software (V.5.3) is used to import data and perform data statistical analysis. For dichotomous data, if *I*^2^ < 50%, then the fixed-effect model will be used for data synthesis, if *I*^2^ is from 50% to 75%, then the random-effect model will be conducted for data synthesis, and if *I*^2^ > 75%, then we will investigate possible reasons from both clinical and methodological perspectives and provide a descriptive analysis or conduct subgroup analysis. On the other hand, for continuous data, if no heterogeneity is detected, the MD or SMD will be used to measure the therapeutic effect of 95% CIs, and if significant heterogeneity is found, then the random-effect model will be used instead.

### 2.11 Subgroup analysis

In case the included studies have high heterogeneity, STATA software will be used to explore the potential sources of heterogeneity, according to different combinations of acupuncture and other therapies, different course time, or different outcome indicators.

### 2.12 Sensitivity analysis

A sensitivity analysis is conducted to assess the stability of results. It is conducted from different aspects including sample size, method quality, and data missing to evaluate its impact on the study. The meta-analysis will be reused and inferior quality research will be excluded.

### 2.13 Grading the quality of evidence

To evaluate the quality of evidence, we used Grading of Recommendations Assessment, Development, and Evaluation (GRADE).^[34]^ However, the downgrading factors include risk of bias, inconsistency, indirectness, imprecision, and publication bias. Moreover, the levels of quality of evidence will be classified into high, moderate, low, and very low.

## 3 Discussion

Cancer is currently the leading cause of death worldwide,^[35]^ and cancer patients are often accompanied by symptoms such as fatigue, pain, depression, and anxiety. The incidence of fatigue symptoms caused by cancer or cancer treatment is about 50% to 90%, which has a great adverse impact on the life and work of cancer patients. Although there are many ways to treat CRF, many patients are not satisfied with their treatment outcomes. Furthermore, acupuncture is considered an important part of traditional Chinese medicine, which has recently been widely used internationally as an adjunct medicine and has a good effect on many diseases. After searching through the literature, it has been found that there have been some studies on the acupuncture treatment for CRF by using a randomized control method. The research results show that acupuncture treatment is effective in treating CRF, but there are inconsistencies in the conclusions drawn by different studies. Although there has been a systematic review of acupuncture treatment for CRF, a large number of new clinical studies on acupuncture treatment for CRF have been found in recent years, so this study aims to systematically evaluate the effectiveness and safety of acupuncture for the treatment of CRF through the past and existing clinical studies. If it is proved that acupuncture treatment for CRF is effective and safe, then we will provide clinicians with new treatment measures and improve the patients QoL.

## Author contributions

**Data curation:** LiJie Tang, ZhengKang Liu.

**Formal analysis:** TaiJun Jiang, FengYa Zhu, LiJie Tang, ZhengKang Liu.

**Investigation:** TaiJun Jiang, FengYa Zhu.

**Methodology:** LiJie Tang, Xi Wu.

**Project administration:** Xi Wu.

**Resources:** ZhengKang Liu.

**Software:** TaiJun Jiang, ZhengKang Liu.

**Supervision:** FengYa Zhu.

**Writing – original draft:** TaiJun Jiang.

**Writing – review & editing:** TaiJun Jiang, FengYa Zhu, Xi Wu.
